# Comparative Study Between Blood Glucose Meters in a Maternity Center

**DOI:** 10.1089/whr.2024.0030

**Published:** 2024-07-26

**Authors:** Rihab Ayadi, Monia Tlig, Imène Ben Jdida, Kaouther Zribi, Linda Khefacha, Mouna Sassi, Balsam Kacem, Amani Chérif

**Affiliations:** ^1^ Faculty of Pharmacy of Monastir, Monastir, Tunisia.; ^2^ Pharmacy Service, Monastir Maternity and Neonatology Center, Monastir, Tunisia.; ^3^ Sfax Hygiene Center, Monastir, Tunisia.; ^4^ Hematology laboratory, maternity and neonatology hospital, Monastir, Tunisia.; ^5^ Pharmacy department, University Hospital Sahloul of Sousse, Monastir, Tunisia.

## Abstract

**Background:**

Monitoring of diabetes by measuring capillary blood glucose using a glucometer.

**Objectives:**

To compare the three most used glucose meters in diabetic patients in a maternity and neonatal center in terms of repeatability, accuracy, and stability.

**Methodology:**

A comparative study on 100 diabetic patients admitted to the various departments of a maternity and neonatal center. For each patient, a capillary measurement was made using each of the three glucometers to be tested (Accucheck^®^, On Call^®^ and Bionime^®^) as well as a blood glucose on venous blood, performed in the laboratory using the Siemens X brand plus^®^ PLC (reference method). The same sample was used to carry out all measurements.

**Results and Conclusions:**

The Accucheck^®^ brand reader and the On Call^®^ brand thus, show a better correlation for open and closed bottles of test strips’storage, compared with the Bionime^®^ brand reader. We found that the Accucheck^®^ and On Call^®^ readers are comparable in terms of reliability of results and are better than the Bionime^®^ brand reader. We have found that, first using closed vials give more accurate results for glycemia readers and also, On Call^®^ and Accucheck^®^ brand readers are more reliable and accurate than the Bionime^®^ brand reader.

## Introduction

Diabetes is defined as a blood glucose level greater than 1.26 g/L (7 mmol/L) after 8 hours of fasting and checked twice.^1^ According to the World Health Organization, the number of people with diabetes increased from 422 million to 532 million between 2014 and 2021, and mortality attributable to diabetes increased by 2.5 million between 2019 and 2021.^2^ Poorly controlled diabetes can cause serious complications that can affect vital organs. For this, having a balanced diabetes is of major importance to delay or even better avoid such complications. Thus, self-monitoring of blood glucose allows regular monitoring of blood glucose and thus better control of diabetes, especially in patients with type 1 diabetes.

Several types of self-monitoring devices are on the market and the objective of our study was to evaluate the reliability of capillary glucose measuring devices used in pregnant women, in terms of accuracy, repeatability, and stability.

## Methodology

This is a comparative study of 100 diabetic pregnant women admitted in a maternity and neonatal center. The study was performed in accordance with the Declaration of Helsinki and was approved by the ethics committee of the Maternity Center. Written informed consent was obtained from all subjects in advance.

The objective of our study was first to compare the three most commonly used glucose meters in diabetic patients in our hospital, by comparing the values obtained by the glucometers to those measured by the autoanalyzer of the biology laboratory of the same center.

For each patient, a capillary measurement was made using each of the three glucometers to be tested as well as a blood glucose on venous blood, performed in the laboratory using the Siemens X pand plus^®^ autoanalyzer. The same sample was used to carry out all measurements. All measurements were performed on whole blood glucose using EDTA (ethylenediaminetetraacetic acid) tubes.

In a second step, in order to verify the reliability of the results according to the storage conditions of the strips, we carried out for each patient two measurements per glucometer, a first with a strip kept in a closed vial and a second with a strip kept outside its storage bottle for 24 hours, in order to mimic the practices of patients or nurses measuring capillary glucose levels in some clinical departments, who leave the strips’ containers open.

Three glucometer brands were used in this study: Bionime GM550, 101-3GM550-234, BIONIME CORPORATION (reader A), Accuchek Instant, Roche USA—84035 V1/1—09291539061 (01) (reader B) and On Call plus 103L12DA18F (reader C). Two tested blood glucose meters use glucose dehydrogenase-impregnated strips as their assay method. The third one uses strips with glucose oxidase.

The results obtained by glucometer were compared with those obtained after determination of venous blood by the laboratory autoanalyzer using the method of hexokinase-6-phosphate dehydrogenase, which is presented as an accepted reference method for glucose measurement.

The laboratory value is the reference value.

The accuracy of the glucometers was determined by the correlation coefficient between the reference value and the value read on the glucometer.

The formula of accuracy is as follows:

$$\mathrm{Accuracy}=\frac{V_{R}-V_{L}}{V_{R}}*\;100$$

With V_R_: Reference value and V_L_: Value read on the reader.

The different values were expressed in mmol/L.

The standard deviation between the calculated accuracy values was calculated for the different glucometers in open and closed vial situations according to the intervals of the following reference values: <4mmol/L; between 4 and 6 mmol/L; between 6 and 7 mmol/L and > to 7 mmol/L to verify the reliability of the meters in the detection of mild, severe hypoglycemia, or hyperglycemia.

## Results

A total of 600 measurements per blood glucose meter (200 measurements per meter) and 100 measurements on venous blood in the laboratory were performed. Of the 100 measurements values performed with the Siemens X pand plus^®^ autoanalyzer, 9 were <4 mmol/L, 63 were between 4 and 6 mmol/L, 14 were between 6 and 7 mmol/L, and 14 were >7 mmol/L.

The correlation coefficients for the different blood glucose meters are presented in Table 1.

**Table 1.** Correlation Coefficient of Different Glucose Meters

Reader	Bionime^®^ reader	Accucheck^®^ reader	On Call^®^ Player
Closed vials			
Correlation coefficient	0,97	0,98	0.98
Open vials			
Correlation coefficient	0,79	0,86	0,94

The reader of the Accucheck^®^ brand and the On Call^®^ brand thus have a better correlation for open and closed bottles compared with the reader of the Bionime^®^ brand.

**FIG. 1.**
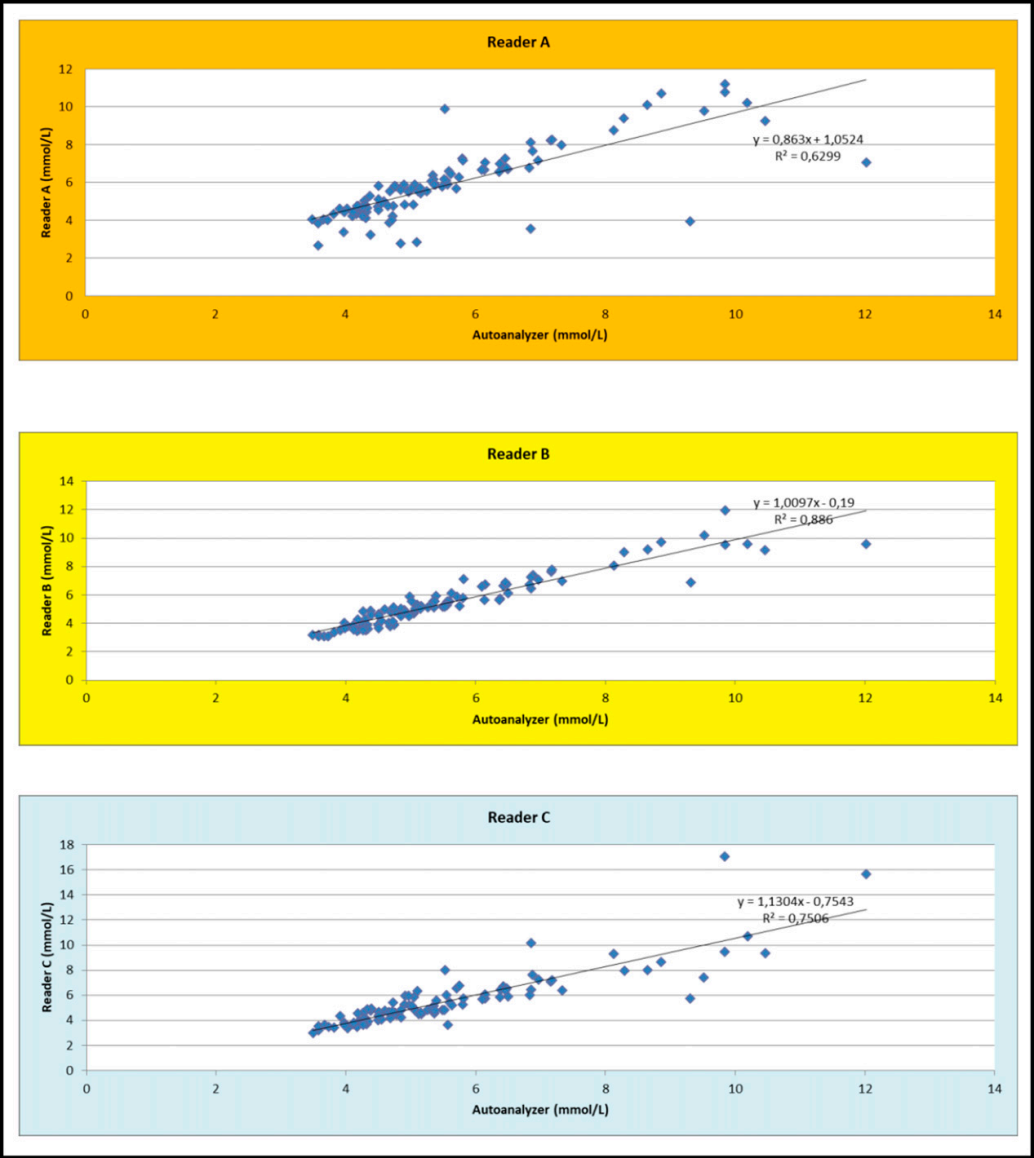
Calculated accuracies for the three glucometers, closed vial (measurements made on the same samples). Reader **(A)**: Bionime^®^, Reader **(B)**: Accucheck^®^, Reader **(C)**: On Call^®^.

**FIG. 2.**
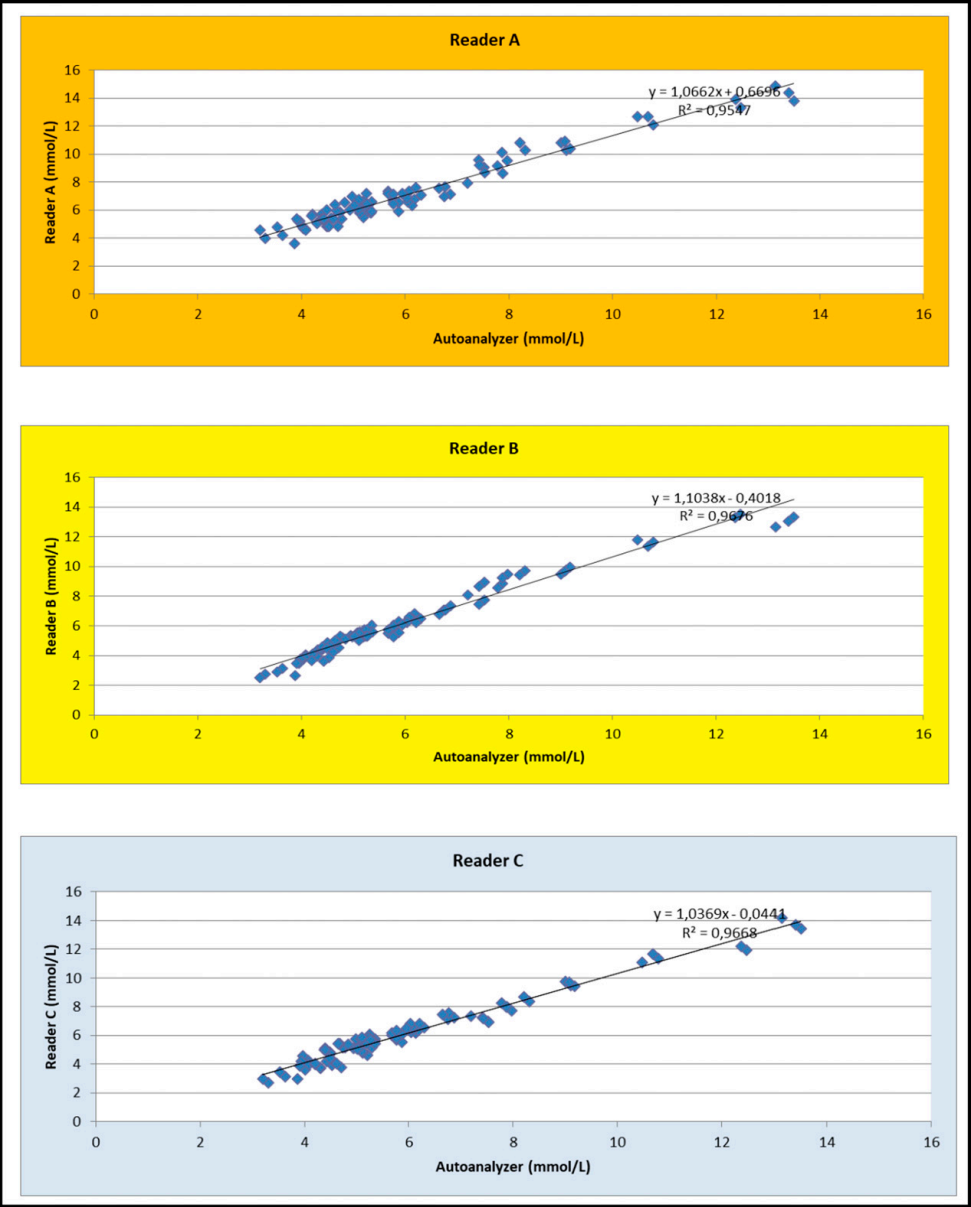
Calculated accuracies for the three glucometers, open vial (measurements made on the same samples). Reader **(A)**: Bionime^®^, Reader **(B)**: Accucheck^®^, Reader **(C)**: On Call^®^.

Figure 1 and Figure 2 show the different accuracies calculated for the three glucometers, when the vials are closed and open respectively. The Accucheck^®^ reader (B) has better reliability, with 76% accuracy at +/− 10% for closed vials versus 72% for the On Call^®^ (C) brand reader and only 20% for the Bionime^®^ reader (A). The values outside the +/− 20% range were 3% for the Accucheck^®^ reader, 2% for the On Call^®^ reader and 46% for the Bionime^®^ reader.

In addition, we note a tendency to overestimate blood glucose by the Bionime^®^ meter, at the risk of masking hypoglycemia.

For the Accucheck^®^ meter, blood glucose is rather underestimated for low blood glucose values. The Accucheck^®^ and On Call^®^ readers are thus comparable in terms of reliability of results and are better than the Bionime^®^ reader.

**FIG. 3.**
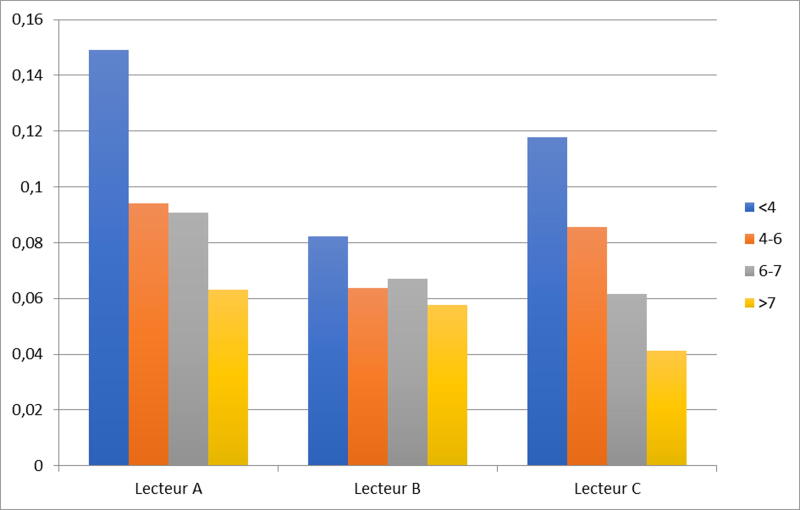
Standard deviation on the accuracies for the 3 meters for closed vials, according to the reference value on automation of the glycemia.

Figure 3 shows the standard deviations on the accuracy of the three readers using closed vials.

This figure shows that for low blood glucose levels, the standard deviation for the Accucheck^®^ meter is lower and for blood glucose levels above 6 mmol/L, the standard deviation for the On Call^®^ meter is smaller. The standard deviation is greater for the Bionime^®^ meter, regardless of the glycemic figures.

Other technical characteristics were evaluated for the three readers: easy reading, display delay, drop deposition by capillarity, sampling size, calibration with plasma, memorization of measurements up to 300 measurements and detection of errors, and high and low limits. They proved satisfactory for all three readers.

## Discussion

The reliability of blood glucose meters is an indispensable element for monitoring blood glucose in diabetic patients, especially insulin-dependent diabetes.^3^

We compared the results of blood glucose measurements between three brands of glucometers, using strips removed from open and closed vials. The difference in results observed between open and closed vials is due to exposure of the strips to oxygen in the air and temperatures outside the range specified by the manufacturer. However, this factor simulates really conditions of use where nurses tend to leave the bottles of the open strips lying around. In addition, interindividual variations in hematocrit, altitude and blood pressure of patients can influence the reliability of measuring devices.^4^

The analysis of standard deviations on the accuracy between the 3-meter marks shows that the lower the blood glucose value, the greater the standard deviation on precision. The latter, decreases when we tend towards hypoglycemia. This could be due to a low sensitivity of the device. It should also be noted that significant errors in the detection of hypoglycemia have been reported with some devices, for example, errors have been reported with the brands BeneCheck PLUS JET,^®^ On Call Advanced^®^, Yuwell Accusure 710^®^ and others.^3^ It should be also mentioned that, as shown in our results, the accuracy of blood glucose meters is better when the strips are stored in closed vials rather than in open vials, and it is important to emphasize the good storage conditions of the strips with patients to ensure better reliability of the readers.

On the other hand, the three brands evaluated have positive linearity when the vials are closed. These values move away from the unit if the vials are opened. This indicates the considerable influence of environmental factors on the accuracy of the strips. The differences in correlation coefficients between the three brands could be due to inappropriate calibration due to improper handling by the user or poor training by the manufacturer.^5^

The results of our study emphasize the importance of the conditions of use and storage of blood glucose meters. In addition, it is important that manufacturers of these devices comply with international standards in terms of performance qualifications to ensure standardization between the different brands existing on the market.^6^ It should also be mentioned that the training of nursing staff on the use of these devices by manufacturers is of considerable importance to ensure the correct use of the glucometer and the correct measurement of blood glucose.

A study conducted in Taiwan testing the performance of the Bionime^®^ meter reported excellent linearity for the measurement of blood glucose (R 2 > 0.99) with an accuracy described as satisfactory (constant variation: 1.1–2.8%) for concentrations between 0.6 and 30.5 mmol/L.

Another study compared the accuracy of 27 blood glucose monitoring systems against a reference method. For the Bionime^®^ brand, they found that for blood glucose levels <4.16 mmol/L, 100% of the measurements deviate at most ±15% from the results of the reference method. In addition, for blood glucose levels > 4.16 mmol/L, 100% of the results are at an interval of ± 20%. Regarding the AccuCheck^®^ brand, they found that for blood glucose levels <4.16 mmol/L, 100% of measurements are within ±15%. In addition, for blood glucose levels ≥4.16 mmol/L, they reported that 100% of the results are within a range of ± 20% of the reference method results.

This study concluded that the performance of Bionime^®^ and Accucheck^®^ meters is satisfactory for normal blood glucose values. The accuracy of these two meters is all the more important as blood sugar decreases. This is consistent with the results of our study.

For the reader of the On Call brand, a study in Ethiopia compared the performance of the On Call^®^ brand with three other brands compared with a reference measurement method that is based on strips impregnated with the enzyme hexokinase. The results obtained show that not all the brands studied are satisfactory either in terms of accuracy or reliability.^7^

The discrepancy between our results and those of the literature could be due to the heterogeneity of study types, the difference in reference methods as well as several types of errors (pre-analytical, analytical, and post-analytical).

## Conclusion

Admittedly, blood glucose meters are not a method of diagnosing diabetes, but their role is essential in the urgent case of patients’ management.^8^ By interpreting the results of our study, the readers of the On Call^®^ and Accucheck^®^ brand are more reliable and accurate than the reader of the Bionime^®^ brand. Since the reference method is different from the method used, we found that the Bionime^®^ brand reader may require technical tune-up. Nevertheless, the three brands of readers in question can be used for the monitoring of diabetic patients in an effective manner. Finally, considering our small sample size of patients, further studies, with a larger sample size and a wider age range, are needed to confirm the reliability of our results.

## Declaration of Submission

All authors certify that the submitted article is original, it is not considered by another journal, it has not already been published, and it has been approved by all authors.

All authors of this article all authors meet the authorship requirements according to the ICMJE international criteria to define authorship.

By submitting this article, I agree to transfer the copyright to the Journal de Women’s Health Reports.

## Links of Interest

All the authors of this article attest that there are no links of interest.

## Abbreviations Used

EDTA: ethylenediamineetracetic acid
R2: the coefficient of determination
Vl: value read of on the reader
Vr: reference value
